# Mapping strategy for multiple atrial tachyarrhythmias in a transplant heart

**DOI:** 10.1186/s12872-015-0031-3

**Published:** 2015-05-12

**Authors:** Qi Jin, Steen Pehrson, Peter Karl Jacobsen, Xu Chen

**Affiliations:** Department of Cardiology, The Heart Centre, Rigshospitalet, University of Copenhagen, Copenhagen, Denmark; Department of Cardiology, Shanghai Ruijin Hospital, Shanghai Jiao Tong University School of Medicine, Shanghai, China

**Keywords:** Atrial arrhythmias, Heart transplantation, Remote magnetic navigation

## Abstract

**Background:**

Different atrial arrhythmias can coexist in the recipient and donor atria after heart transplantation.

**Case presentation:**

We report an unusual case of a patient with three different types of atrial arrhythmia after heart transplantation: an atrial fibrillation in the recipient atria, and a cavotricuspid isthmus dependent atrial flutter and a focal atrial tachycardia in the donor atria. 3D electroanatomical mapping and ablation were guided by remote magnetic navigation (RMN). Atrial fibrillation continued in the recipient atria even after the donor heart was converted to sinus rhythm by ablation.

**Conclusions:**

It is critical to understand the surgical anatomy of a bi-atrial anastomosis and its relevant electrical activation pattern before ablation. Appropriate electroanatomical mapping strategy with RMN can facilitate the successful ablation of post-transplant atrial arrhythmias.

## Background

Although the incidence of atrial arrhythmias after heart transplantation has been decreasing in the past decades, it has still ranged from 10 to 20 % in recent studies [1, 2]. Different supraventricular rhythms can coexist in the recipient atria and donor atria [3–5]. Better understanding of the surgical anatomy of a bi-atrial anastomosis and the electrical activation patterns by 3D electroanatomical mapping allowed us to identify the mechanisms of different atrial arrhythmias, and thus, approach their successful ablation [5]. Here, we reported an unusual case of a patient with three different types of atrial arrhythmia after heart transplantation: an atrial fibrillation (Af) in the recipient atria, and a cavotricuspid isthmus (CTI) dependent atrial flutter (AFL) and a focal atrial tachycardia (AT) in the donor atria.

## Case presentation

**Fig. 1:**
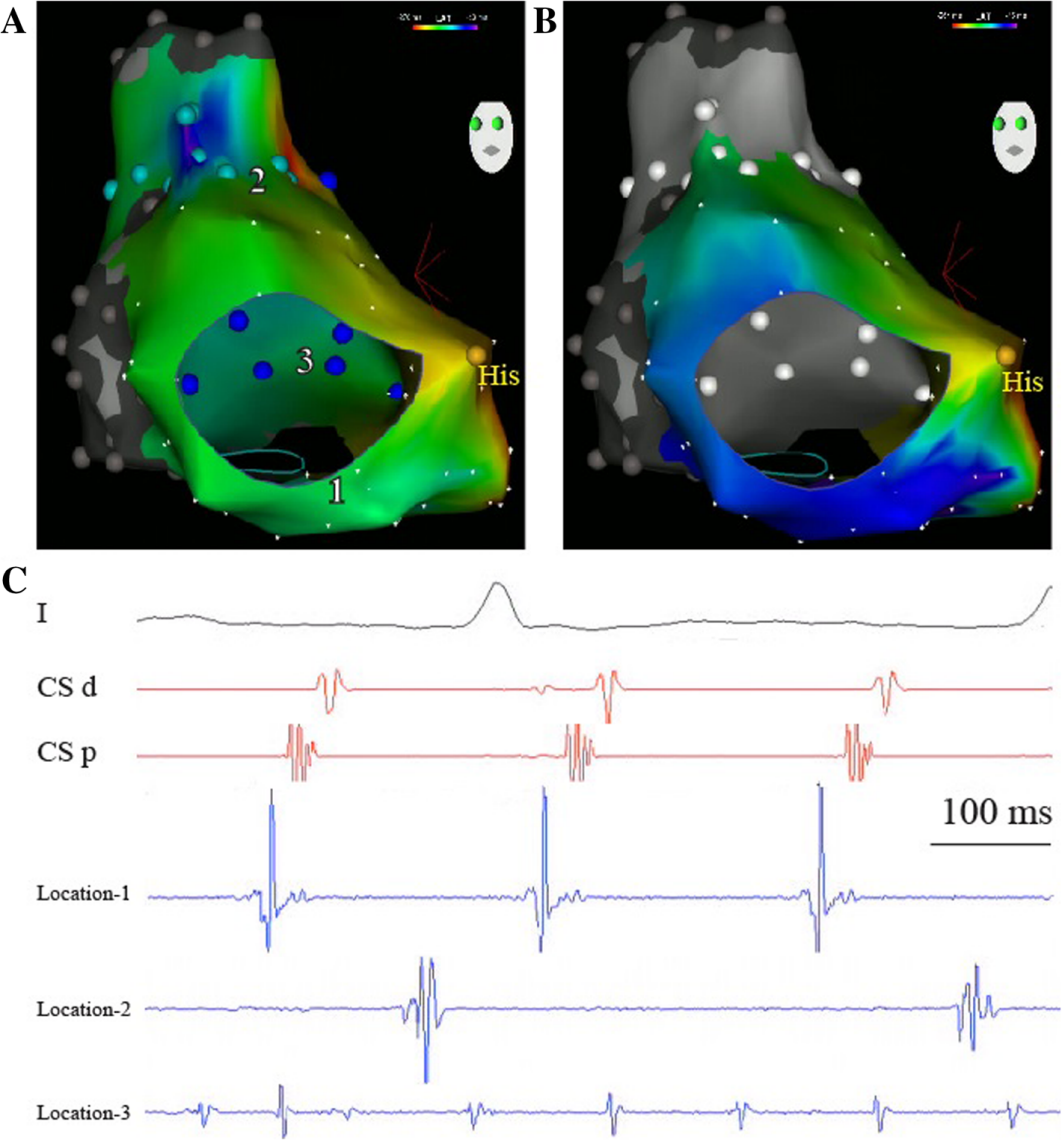
Panel **A** shows a left anterior oblique (*LAO*) view on a CARTO activation map in the recipient and donor atria. The *red color* represents the earliest activation. The *light blue points* represent the sites of 2:1 conduction block at the border zone. The *blue points* represent the sites with Af activation in the recipient atria. The *grey points* represent scar areas. In Panel **B**, only activation mapping of the clinical AFL in the recipient RA is performed. The activations of Af and 2:1 conduction are both tagged only by location (*white points*). A counterclockwise CTI-dependent AFL is present in this modified activation map. Panel **C** shows the different activation electrograms of the ablation catheter at the different sites. Location *1* is at six clock relative to the TA. Location *2* and *3* are at the anterior-superior wall and posterior-inferior wall respectively

**Fig. 2:**
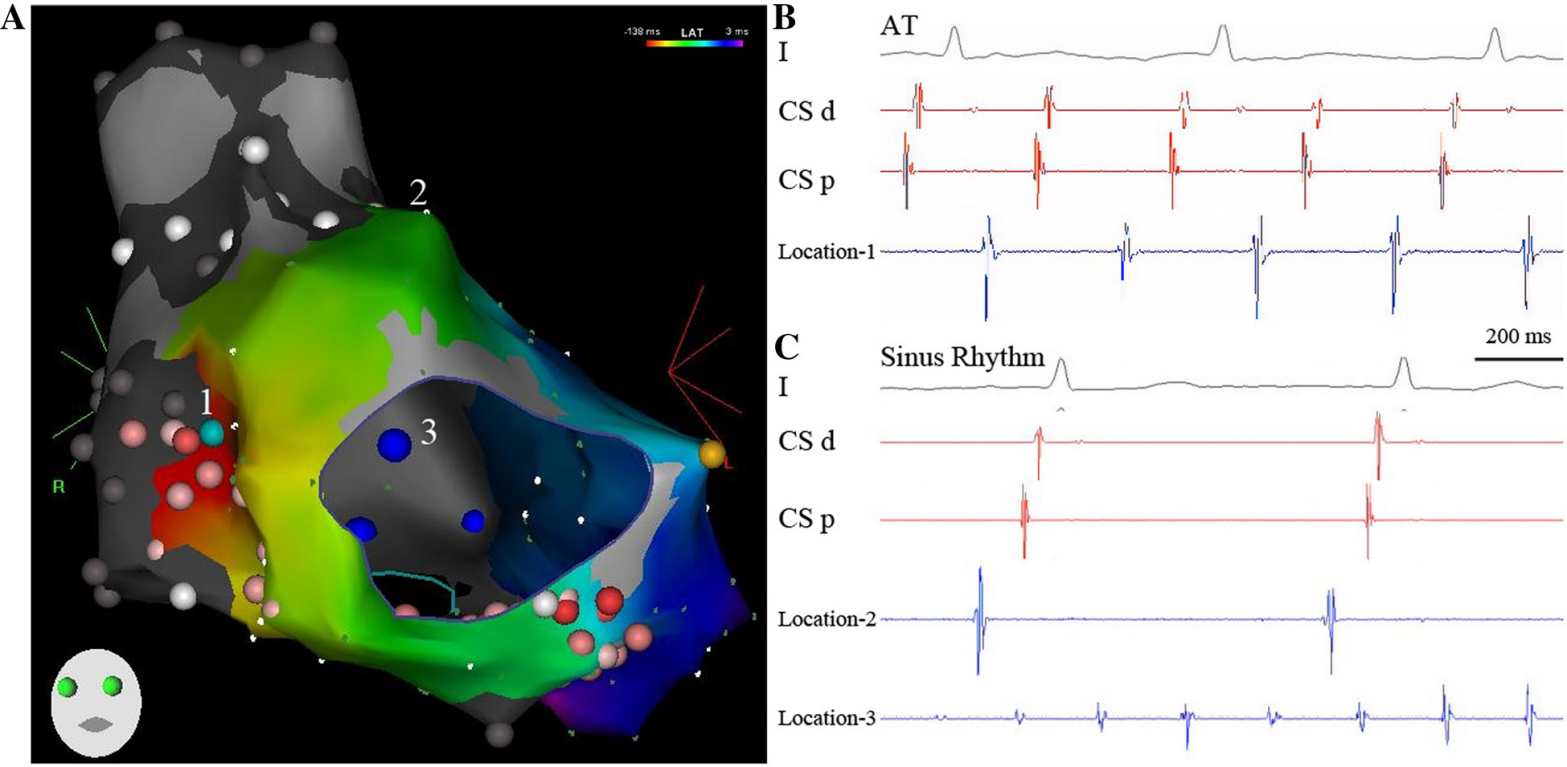
In Panels **A** and **B**, activation mapping from the donor atria indicates an origin of a focal *AT* at the suture line (border zone, location *1*). The *red color* represents the earliest activation. At this site, radiofrequency current is applied and terminates *AT. Red* and *pink points* indicate the ablation areas. The color’s strength represents the duration of ablation delivery. Panel **C** shows the patient’s sinus rhythm with a cycle length of 800 ms. The ablation catheter at location *2* (anterior-superior wall of the donor atrium) indicates *sinus rhythm*. Af at the ablation catheter in the recipient atria (*blue point*, location *3*) is not changed, but the two atria are electrically dissociated

A 65-year-old man underwent orthotopic heart transplantation with bi-atrial anastomosis due to dilated cardiomyopathy in 1995. The patient developed symptomatic persistent AFL that was refractory to anti-arrhythmic drugs for 6 months before the procedure. The surface ECG showed a positive P wave in lead V1 with P-P interval of 230 ms, and 2:1 relation of P/QRS. After the patient signed an informed consent, an electrophysiological study was performed to elucidate the mechanism of the AFL. A 6F steerable catheter (Inquiry, St Jude Medical, Inc., Irvine, CA, USA) and a 5F quadripolar catheter (Medtronic, Inc., Minneapolis, MN, USA) were positioned within the coronary sinus (CS) and at the apex of right ventricle via the left femoral vein, respectively. CS activation pattern and entrainment mapping from CS indicated the clinical AFL originating from right atria (RA) (Fig. 1C). An open irrigated magnetic ablation catheter (Navistar Thermocool-RMT, Biosense Webster Inc., Diamond Bar, CA, USA) was used to perform 3D RA electroanatomic mapping and ablation by the CARTO RMT system and the remote magnetic navigation system (RMN, Stereotaxis Inc., St. Louis, MO, USA). During the initial activation mapping, four different activation patterns coexisted in the donor and recipient atria: 1) AFL with a cycle length of 230 ms at the anterior and septal wall of the donor atria; 2) Af in the recipient atria; 3) scar tissue between the donor and recipient atria; 4) 2:1 conduction from AFL at the zone between AFL area and scar area (Fig. 1). An activation map containing of the donor and recipient atria led to the complexity of understanding the mechanism of the clinical AFL (Fig. 1A). Thus, we performed the activation mapping only focusing on the AFL in the recipient RA by defining all other areas as scar or location only. A counterclockwise CTI-dependent AFL in the donor atria appeared in the modified activation map (Fig. 1B). Linear ablation guided by RMN was performed at the inferior wall between the atrial suture and the tricuspid annulus (TA). After termination of AFL, an AT with the cycle length of 310 ms was induced in the donor atria by atrial stimulation. A re-map showed a focal AT, which was converted to sinus rhythm by ablation at the focal existence at the border zone between recipient and donor atria (Fig. 2). Af in the recipient atria still continued, indicating that the two atria were electrically dissociated (Fig. 2). The fluoroscopy time was 3 min. No complications occurred during the procedure.

## Discussion

Post-transplant atrial arrhythmias were most commonly due to atrial macro-reentry including CTI-dependent and scar-related circuits [1, 6, 7]. Focal AT originating in low-voltage or border zones adjacent to the bi-atrial anastomosis was another possible mechanism [8]. In this case, these two different mechanisms of atrial arrhythmias were detected in the donor atria. Similar to typical AFL in non-transplanted patients, the reentrant circuit of the clinical AFL in this case was around the TA (Fig. 1B). Differently, the ablation line was designed to be relatively narrow for the active component of the isthmus from the TA to the atrial suture line rather than to the inferior vena cava (Fig. 2). Radiofrequency energy can achieve isthmus block. An ectopic focus at the border zone induced a non-clinical AT in the donor atria, which was also successfully ablated. This case indicated the importance for the operator to realize that multiple electrical mechanisms can coexist in a transplant heart with bi-atrial anastomosis.

Mapping strategy in the evaluation of atrial arrhythmias in a transplant heart is a key factor to achieve successful ablation [3]. Activation mapping requires generation of complete and dense maps and correct annotation of electrograms may be challenging, especially when activation of the entire atria continued more than the cycle length of the tachycardia [9]. As described above in this case, coexisting donor AFL, recipient Af and the zone with 2:1 AFL conduction made the initial activation mapping strategy of the entire atria impossible to explore the mechanism of the clinical AFL (Fig. 1A). Better understanding the surgical anatomy of bi-atrial anastomosis and its possible electrical correlates helped us to adjust the activation mapping strategy, and thus, elucidate the real mechanism.

Of note, this case was performed by RMN. Mapping and ablation using RMN may offer advantages during some complex procedures compared to manual techniques [10]. RMN systems can help the operator to manipulate the catheter to map the critical regions with high density and complete the designed ablation line in a transplant heart with relatively shorter fluoroscopy time (3 min in this case).

Many factors could be related to atrial tachyarrhythmias after heart transplantation. One of limitations in this study is that we cannot rule out the role of previous transplant rejection or chronic vasculopathy because we did not perform the biological test before or after the ablation procedure.

## Conclusions

To our knowledge, it’s the first report for a heart transplant patient in whom these two different mechanisms of atrial arrhythmias were observed in the donor atria. Appropriate electroanatomical mapping strategy with RMN may facilitate successful catheter ablation for the multiple atrial tachyarrhythmias in a transplant heart.

## Consent

Written informed consent was obtained from the patient for publication of this case report and any accompanying images. A copy of the written consent is available for review by the Editor of this journal.

## Abbreviations

Af: Atrial fibrillation
AFL: Atrial flutter
AT: Atrial tachycardia
CTI: Cavotricuspid isthmus
TA: Tricuspid annulus
RMN: Remote magnetic navigation
